# Superficial Temporal Artery Island Flap Combined With Laser Hair Removal for Inadequate Skin Expansion Following Tissue Expansion: A Case Report of Congenital Melanocytic Nevus of the Forehead in an Adult

**DOI:** 10.7759/cureus.55920

**Published:** 2024-03-10

**Authors:** Toshifumi Yamashiro, Momoko Yoshida, Junki Sato, Ryuichi Azuma

**Affiliations:** 1 Department of Plastic and Reconstructive Surgery, National Defense Medical College, Tokorozawa, JPN

**Keywords:** surgical planning, tissue expander, complications, reconstructive surgery, flap surgery, superficial temporal artery flap, congenital melanocytic nevus, tissue expansion

## Abstract

Tissue expansion is a handy reconstructive technique for the head and neck region; however, its implementation requires careful planning and surgical experience. If tissue expansion is inadequate, forced closure results in wound tension and risks complications, such as postoperative deformity, wide scarring, and wound dehiscence. We report a case of adult forehead melanocytic nevus excision using a tissue expander (TE) where complications caused by insufficient tissue expansion were avoided by creating a flap using a dog ear. The patient was a male in his 20s who underwent surgery with a TE for a congenital melanocytic nevus sized 15 × 10 cm on the left forehead. Resection was performed by tissue expansion using two TEs; however, simple advancement flaps led to excessive wound tension, risk of elevation of the eyebrow on the affected side, and postoperative scarring. Hence, a superficial temporal artery fasciocutaneous island flap with left superficial temporal vessels as a pedicle was raised at the dog ear and moved to the site of strong tension, and the wound was closed without difficulty. Although postoperative laser hair removal was required, both the appearance and functional results were satisfactory. Using anatomical flaps obtained from the surroundings during tissue expansion helps avoid complications associated with forced wound closure.

## Introduction

Tissue expansion is a useful reconstructive technique for the treatment of tissue defects resulting from trauma or surgery [1]. A simple suture is chosen if the tissue defect is small; however, if it is large, the tissue must be replenished. Tissue expansion stands out due to its utilization of expanded tissue around the defect, offering advantages such as reduced susceptibility to color-match and texture-match anomalies resulting from variations in donor and recipient anatomy - common concerns with skin grafts, distant flaps, and free flaps. Furthermore, tissue expansion mitigates geometric scarring associated with certain local flaps. However, tissue expansion has the disadvantage of being a two-stage procedure, which may cause cosmetic problems during the implantation period depending on the implantation site, the size of the tissue expander (TE), and the risk of complications associated with the long-term implantation of a TE, which is an artificial material [2,3].

Also, surgeons must be experienced in determining the size and shape of the TEs, the number of TEs, and their placement, and may encounter difficulties during reconstructive surgery when tissue expansion is not satisfactory [4,5]. For reconstruction of the forehead, tissue defects occupying 25-70% of the area are considered suitable for tissue expansion, while smaller defects may be addressed through serial excision and larger ones through skin grafting [6]. We resected an adult congenital pigmented nevus of the forehead using two TEs, which resulted in lesser-than-expected tissue expansion. To avoid postoperative deformities, we created a fasciocutaneous flap using the expanded tissue and achieved successful results, although additional laser hair removal was required.

## Case presentation

**Figure 1:**
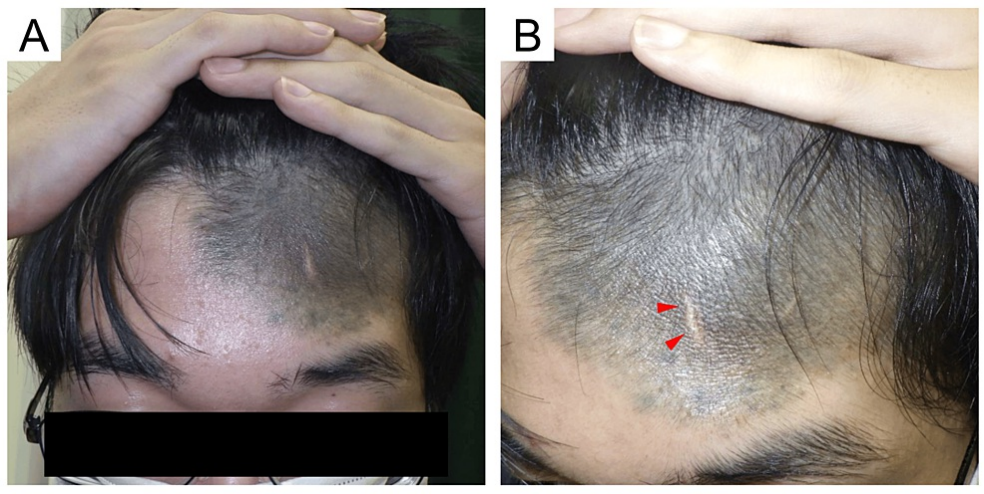
Clinical photographs at the time of initial examination (A) Frontal view: a hairy congenital melanocytic nevus sized 15 × 10 cm is observed on the left forehead beyond the midline and hairline. (B) Oblique view: a scar from a tissue biopsy in childhood is observed in the center of the lesion (red arrowhead)

A 24-year-old male with no other comorbidities was referred to our hospital for the treatment of a congenital melanocytic nevus on the left frontal area of the head. Upon initial examination, a 15 × 10 cm large hairy melanocytic pigmented lesion was observed extending from almost the entire left forehead to the temporoparietal area of the patient (Figure 1A). He had been diagnosed with a congenital melanocytic nevus by tissue biopsy at an early age (Figure 1B). The extent of the lesion precluded sequential excision due to its size, occupying more than 50% of the frontal area [6]. Additionally, given the tumor's location beyond the hairline, complete excision coupled with skin grafting was anticipated to lead to alopecia. Consequently, we decided to perform resection using TEs, and six months following the initial consultation, the TEs were surgically implanted in the first procedure performed under general anesthesia.

**Figure 2:**
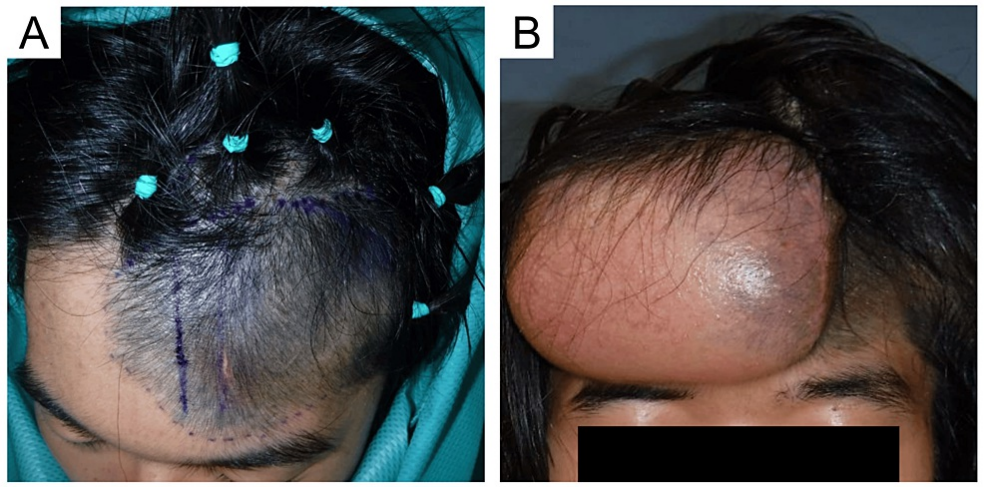
Photographs before and after the initial surgery (A) Two rectangular-type TEs (KOKEN, Tokyo, Japan) were inserted. The first TE (290 cc, 100 × 50 × 70 mm) was inserted under the frontalis muscle on the healthy side through a longitudinal incision in the nevus, and the second TE (330 cc, 140 × 70 × 40 mm) was inserted under the epicranial aponeurosis through a hairline incision in the nevus. (B) Clinical photograph after the completion of expansion TE: tissue expander

Two rectangular TEs (KOKEN, Tokyo, Japan) were inserted during the initial surgery. The first TE (290 cc, 100 × 50 × 70 mm) was inserted under the frontalis muscle of the healthy side following a longitudinal incision within the nevus, and the second TE (330 cc, 140 × 70 × 40 mm) was inserted under the epicranial aponeurosis following a hairline incision within the nevus (Figure 2A). The TE was inflated by 10% on an outpatient basis over approximately three months, and the nevus was resected as the second operation one month after the expansion of the TEs to their maximum capacity (four months after the initial operation) (Figure 2B).

**Figure 3:**
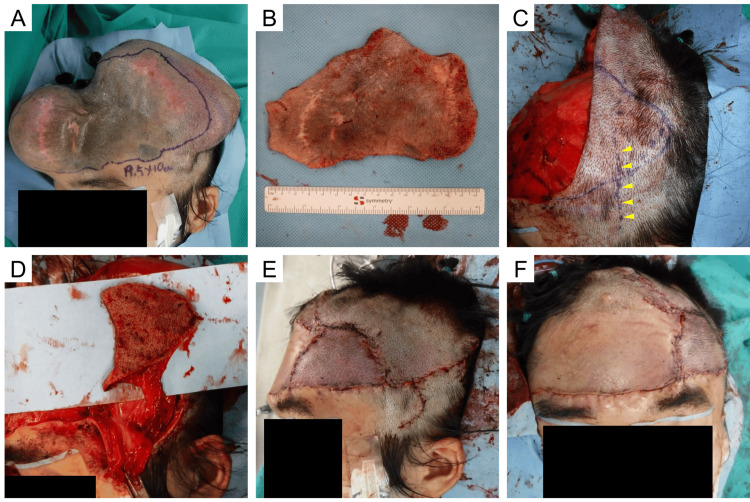
Intraoperative photographs of the second surgery TE removal and nevus excision were performed four months after the first surgery and one month after expansion completion. (A) The resection area was set with a horizontal margin of 2 mm from the nevus edge. Part of the nevus was expanded, and the resection area was determined to be 19.5 × 10 cm in size. (B) The excised nevus. (C) STA and its branches were identified using handheld Doppler ultrasound (yellow arrowhead), and the STA island flap was designed at the area of the dog ear caused by the posterior expanded flap advancement. (D) The STA island flap pedicled with STA, concomitant vein, and surrounding temporoparietal fascia was elevated and moved to the forehead. (E) Lateral view immediately after the surgery. The STA flap placement allowed all sutures to be placed without excess tension. (F) Frontal view immediately after the surgery. The flap allowed wound closure without elevation of the eyebrows TE: tissue expander; STA: superficial temporal artery

During the second surgery, the nevus was resected en bloc under the frontalis muscle and epicranial aponeurosis with a horizontal margin of approximately 2 mm. A certain portion of the nevus was expanded, and the resected area was 19.5 × 10 cm in size (Figures 3A, 3B). When the TE was removed and the margins of the expanded skin flaps were cut and lengthened, the tissue extension was sufficient to allow closure of the defect; however, high tension of the sutures between the lengthened flaps caused traction and elevation of the left eyebrow, which would result in a prominent postoperative scar on the forehead. Therefore, the superficial temporal artery (STA) and its branches were identified via handheld Doppler ultrasound. The STA island flap pedicled with the STA, concomitant vein, and surrounding temporoparietal fascia was elevated using the dog ear caused by posterior flap advancement and moved to the forehead skin defect (Figures 3C, 3D). The placement of the STA flap enabled all sutures to be placed without excess tension or elevation of the eyebrows (Figures 3E, 3F). Postoperative histopathological analysis of the resected tissue revealed a melanocytic nevus without malignancy.

**Figure 4:**
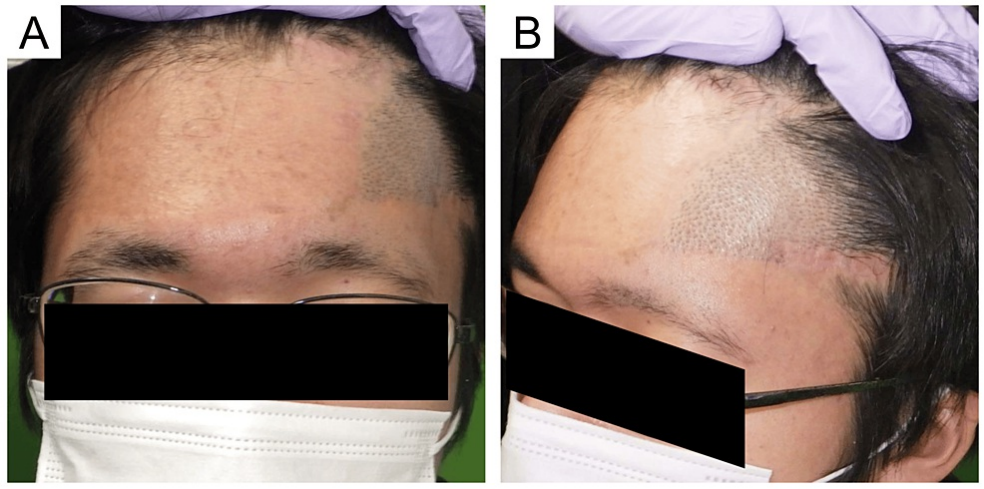
Clinical photographs at six months after nevus excision (A) Frontal view. (B) Oblique view

**Figure 5:**
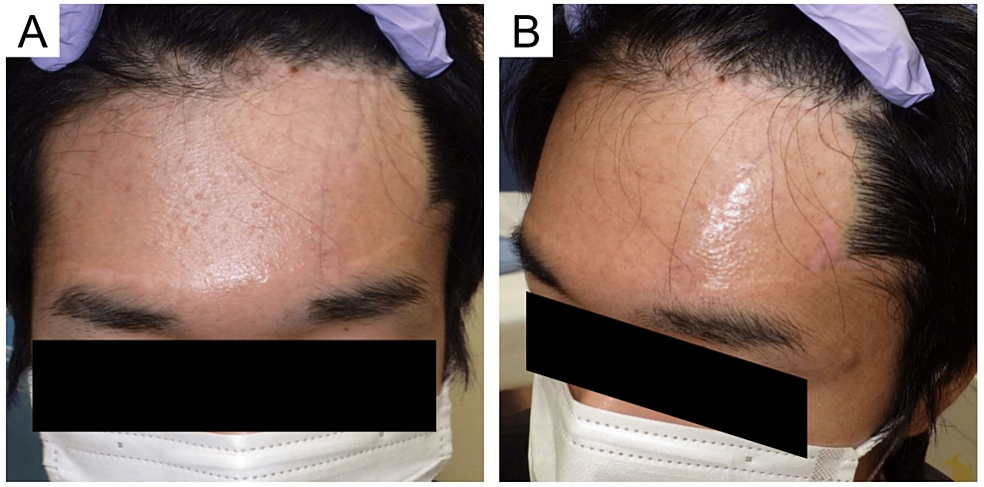
Clinical photograph at 18 months after nevus resection A scar revision operation and laser hair removal with an 810-nm diode laser were conducted. The scar was relatively unnoticeable and the patient was satisfied with the results. (A) Frontal view. (B) Oblique view

The patient experienced no major postoperative complications (Figures 4A, 4B). Eight months after the second surgery, a scar revision surgery involving the removal of scars around the flap margins, hairline, and relatively wide scars in the hair-bearing area was performed, and laser hair removal on the STA flap was performed using an 810-nm diode laser. More than 18 months after the last surgery, a natural appearance to the patient's satisfaction was achieved (Figures 5A, 5B).

## Discussion

When surgical treatment is chosen for melanocytic nevi in adulthood, simple excision is often preferred for small-sized nevi, and serial excisions are often chosen for nevi that are too large to be removed by a single excision [6]. There is a limit to the size of nevi that can be treated by serial excision, and it is only suitable for cases with multiple, relatively small nevi or areas where the surrounding skin is easily developed [7]. Treatment with skin grafts or TEs is necessary in cases difficult to treat via serial excisions. In our case, the patient had a medium-sized congenital melanocytic nevus measuring 15 × 10 cm, which did not constitute a giant congenital melanocytic nevus (GCMN, defined as a nevus that covers more than 1% of the body surface area in childhood and exceeds 20 cm in diameter in adulthood) [6,8]. The nevus was present beyond the hairline, and reconstructive surgery with TE was planned to avoid baldness associated with skin grafting following total resection.

Reconstructive surgery with TE is widely used for breast reconstruction as well as for tissue reconstruction after trauma and removal of tumors or scars. Tissue expansion is a useful reconstruction method because the tissue surrounding the defect can be expanded and used for reconstruction, and thus it matches well in color, tone, and texture. However, it is known to have a high complication rate [2,9,10]. The most common complication is exposure of TE, while others include infection, rupture, migration, and wound dehiscence [9,10]. The major complication rate is particularly high in the cranial region, as in the present case, probably because of anatomical characteristics such as the presence of the skull and poor tissue extensibility [9].

Appropriate surgical planning is essential for reconstruction using TEs. Careful planning of the size, shape, and position of insertion is necessary [4]. Although tissue defects can be covered by advancing or rotating the TE-expanded flap, insufficient tissue extension occasionally causes excessive tension at the wound edge [5]. Excessive wound edge tension increases the risk of postoperative wound dehiscence, deformity, and wide scars. Hence, studies are underway to improve the utilization of the extended flaps. Using finite element analysis, Buganza-Tepole et al. reported that the efficiency of flap elongation and the location and direction of tension differed depending on the incision method [5]. Ueda et al. reported a method for making a lambda-shaped incision at the site of dilation [11], and Kiyokawa et al. developed and reported a method for deploying dilated skin as a cube [12]. In this case, the most orthodox design of an advancement flap was initially used; however, it was believed to increase the risk of a wide postoperative scar owing to the strong tension at the suture site and traction and elevation of the eyebrow on the affected side. Therefore, the left STA and its branches were identified, and an STA fasciocutaneous island flap was harvested to address the issue.

The STA flap is a flap pedicled with the STA and its concomitant veins. It can be harvested as a temporoparietal fascial flap, hairy cutaneous flap, or temporoparietal osteofascial flap [13-15]. In this case, an STA island flap was created at the dog ear, which was excessive in the advancement of the posteriorly expanded flap, and repositioned to the affected forehead site where the tension was strongest, thereby relieving tension at all sutures and allowing effortless closure. By reconstructing along the aesthetic subunit, it enabled aesthetically pleasing reconstruction [16]. Although this flap was hairy and required postoperative laser hair removal with an 810-nm diode laser, the scar was hidden by the hair, and the results were satisfactory in terms of appearance and function compared with skin grafting. Good results have been reported when tissue expansion is combined with laser hair removal in the head and neck region [17-19]. Although careful preoperative planning is a prerequisite, a combination of flaps may be a useful option when facing difficulties during tissue expansion surgery.

## Conclusions

We described a case where tissue expansion surgery was performed for congenital pigmented nevi of the forehead in an adult by using an STA island flap with dog ears to overcome the lack of tissue expansion. The use of flaps instead of forcible wound closure prevented postoperative deformity and yielded favorable outcomes. The combination of flaps in tissue expansion may be a useful alternative to reduce postoperative complications associated with forced closure.
